# Supplementary material for: Transversal Competencies in Operating Room Nurses: A Hierarchical Task Analysis
Source: Nurs Rep. 2025 Jun 3;15(6):200. doi: 10.3390/nursrep15060200 (PMC12196087; doi:10.3390/nursrep15060200)
Supplement: Supplementary file 1 [file nursrep-15-00200-s001.zip › nursrep-3653120-supplementary.pdf]

Supplementary material

Transversal Competencies in Operating Room Nurses: a Hierarchical Task Analysis

Francesca Reato1, Dhurata Ivziku 2*, Marzia Lommi 3, Alessia Bresil 4, Anna Andreotti 5, Chiara D'Angelo 6, Mara Gorli 6, Mario Picozzi 7 and Giulio Carcano 8,9

Table S1. Example of how observed behaviors are categorized and abstracted in sub-competencies and transversal competencies.

Example	Activity observed	Significant words (codes)	Sub-Competencies	Competencies
1	Managing the delay of the first patient, reorganizing the surgical session, calling the second patient, coordinating with the healthcare assistant (OSS) and the anesthesia nurse, preparing the new set of materials, and optimizing time to prevent further delays and work overload.	planning, organization, anticipation, forecasting, optimization, time management, workflow, prioritization, resource allocation	Task Management, Time Management, Organization, Anticipatory Thought	Task and Time Management
2	Independent search for the missing blade, prompt involvement of the anesthesiologist, evaluation of alternatives, and resolution of the issue through modification of the anesthetic technique	problem, solutions, unforeseen events, alternatives,	Problem Solving, Error Management, Decision-Making	Problem Solving and Decision-Making

		risks, decision-making, evaluation, responsibility, promptness		

Table S2. Summary of total findings of the Hierarchical Task Analysis.

Competencies N = 15	Sub-Competencies N = 50	Sub-Competencies Frequency N = 232	Tasks/Activities N = 630	Tasks/Activities included in the Repertoire
Communication and interpersonal relationship	4	29	57	12
Situation awareness	3	23	56	11
Teamwork	3	15	38	10
Problem Solving and Decision-Making	3	26	79	11
Self-Awareness	2	14	45	11
Coping with Stressor	3	12	43	11
Resilience and Fatigue Management	4	15	31	11
Leadership	4	13	55	11
Coping with Emotions	4	20	37	9
Task and Time Management	4	16	53	9

Ethical and sustainable thinking	5	21	65	12
Adaptation to the context	4	9	18	9
Critical Thinking	2	8	29	10
Learning through experiences	3	7	13	8
Data, Information and Digital Content Management	2	4	11	8

Table S3 Transversal Competencies for Perioperative and Perianesthesialogical Operating Room Nurses.

N	Competencies	Sub-competencies	TASKS/ACTIVITIES
1	Communication and interpersonal relationship	1.1 Communication<br>1.2 Conflict management<br>1.3 Assertiveness<br>1.4 Relationship	Transmit clear, concise, and complete information using verbal, paraverbal, and non-verbal communication to ensure clarity and understanding.<br>Actively listen to the surgical and anesthesiology team, integrating feedback to improve collaboration and team effectiveness.<br>Align expectations among all team members regarding the progress of the surgical procedure and anesthetic management to ensure effective coordination.<br>Keep all team members informed about the next steps of the surgery, ensuring continuous and timely communication.<br>Adapt language to different stakeholders, using congruent facial expressions and body language to avoid misunderstandings.<br>Provide the team, patient, family members, or caregivers with accurate updates during the surgical and anesthesiological process to ensure transparency and involvement.<br>Transmit all necessary information to colleagues, using appropriate tools for precise and timely communication.<br>Use tools to understand the thoughts and feelings of patients, personalizing communication approaches to improve care.

			Verify that team members have understood the messages and information shared, through requesting confirmation or feedback.<br>Coordinate team communication effectively, ensuring a continuous and organized flow of information throughout the procedures.<br>Conduct a continuous and structured process of information collection, ensuring every team member has the necessary data to make informed decisions.<br>Address emergency situations with direct and timely communication, ensuring quick responses from the team, patients, family, and caregivers, while maintaining a calm and reassuring approach..
2	Situation awareness	2.1 Situation awareness<br>2.2 Focus<br>2.3 Attention to detail	Predict potential complications, anticipate future needs, speculate on possible issues, and reduce response time for timely intervention<br>Consider the habits, customs, and practices of operators when acting<br>Preparing the body for relevant stimuli by activating a general alert state<br>Recognize and direct attention toward relevant stimuli, managing conflicting inputs, shifting focus between tasks when needed, and maintaining awareness of multiple concurrent stimuli.<br>Activate control abilities, including visual-perceptive, visuo-spatial, and mnemonic skills, to enhance monitoring and response capabilities in dynamic environments<br>Identify and monitor risks, environmental changes, and patient conditions, ensuring safety while hypothesizing, prioritizing, and organizing tasks based on urgency and complexity<br>Manage available resources effectively, taking responsibility for decisions, actions, and omissions in perioperative and perianesthetic care while adapting to changing circumstances<br>Prepare for necessary procedural adjustments, simplify workflow management, and gather and analyze information to respond promptly to evolving situations<br>Recognize and understand situational dynamics to anticipate actions, make future projections, and provide previews of potential developments based on available data.<br>Observe the entire operating room with a 360-degree view, paying close attention to patient monitoring signals, safety parameters, and surgical field activities.<br>Focus on team needs to proactively anticipate required actions, ensuring efficiency while perceiving and analyzing environmental, temporal, and spatial elements for informed decision-making.

3	Teamwork	3.1 Teamwork<br>3.2 Coordination<br>3.3 Interprofessional collaboration	Respect the roles, competencies, and responsibilities of all team members, promoting inclusiveness, teamwork, and mutual support to enhance collaboration.<br>Delegate tasks effectively, ensuring equitable distribution of activities, resources, and responsibilities while considering specific objectives and team dynamics.<br>Give clear and precise instructions to the team, responding appropriately to colleagues' requests and facilitating cooperation among team members.<br>Manage group dynamics efficiently, using strategies to optimize performance, enhance teamwork, and create a supportive working environment.<br>Utilize the unique and complementary skills of all team members to achieve desired results, ensuring safe and effective perioperative and anesthetic management.<br>Demonstrate dynamism and active participation, fostering engagement and maintaining an inclusive and collaborative team culture.<br>Coordinate activities while considering the actions of others, ensuring smooth workflow and effective teamwork among nursing staff and interdisciplinary professionals.<br>Support colleagues in activity coordination, helping achieve common objectives through shared responsibilities and mutual assistance.<br>Work effectively within an interprofessional team, considering each profession's competencies and collaborating to ensure patient safety.<br>Enhance team performance by applying effective coordination strategies, improving workflow efficiency, and reinforcing cooperative professional relationships.
4	Problem Solving and Decision-Making	4.1 Problem Solving<br>4.2 Error management<br>4.3 Decision-Making	Perceive the problem clearly, identify creative and alternative solutions, and recognize multiple potential resolutions to explore all options.<br>Use strategic reasoning to critically assess the context and evolution of the situation, and reconstruct events for deeper analysis.<br>Formulate and evaluate hypotheses for resolving issues, applying solutions that best address the situation at hand.<br>Work with available resources, find different alternatives, and monitor the chosen solution to ensure its effectiveness.

			Handle unforeseen situations calmly, prevent potential problems by anticipating risks, and prepare for unexpected challenges in advance.<br>Resolve problems quickly and efficiently, analyze complex situations, and identify the root causes of issues to address them effectively.<br>Apply safety protocols consistently to prevent errors, ensuring a fault-free environment in both surgical and anesthesiological practices.<br>Identify situations that require decision-making, evaluate available options thoroughly, and assess the risks associated with each choice.<br>Gather relevant information quickly to make fast, informed decisions, and then implement those decisions precisely.<br>Reassess decisions made and engage in reflective practice, both individually and as a team, supporting choices with evidence-based arguments.<br>Take full responsibility for decisions, consider all consequences before acting, and avoid hesitation that could compromise patient safety
5	Self-Awareness	5.1 Self Awareness<br>5.2 Self Efficacy	Recognize your own limits and the consequences of acting without the necessary competence, ensuring safe and competent care by seeking help or appropriate support when needed, thus fostering a professional growth environment.<br>Demonstrate commitment to self-reflection and self-care, adopting practices for physical, mental, and emotional well-being to prevent burnout, maintain balance, and enhance both personal and professional effectiveness.<br>Know and continually reflect on your short, medium, and long-term goals, aligning personal aspirations with professional development while remaining independent of external influences and challenges, facing obstacles with resilience.<br>Be aware of your ways of acting and reacting, recognizing implicit biases and the impact of your behavior on the surgical and anesthesia team, as well as on the assisted person, to foster an inclusive and patient-centered environment.<br>Recognize your talents, valuing strengths as resources for the development of advanced nursing skills, while also identifying skill gaps and areas for improvement to ensure safe and competent care, particularly in complex situations.

			Accept constructive feedback, distinguishing it from personal opinions, and act with the awareness that personal moral opinions can influence behaviors in the operating room, maintaining a professional and open-minded attitude.<br>Act with the awareness that maintaining a clear understanding of roles and professional boundaries is essential for effective collaboration among healthcare providers, while also being aware of the elements that may create barriers between professionals and assisted persons, promoting respectful and safe interactions.<br>Seek help or appropriate support when necessary, demonstrating transparency and humility in recognizing your own limits and requesting assistance when needed to ensure patient safety and professional accountability.<br>Mobilize self-regulation and self-reflection skills, generating new capacities for thought and action, adapting to various challenges, and strengthening team dynamics through a proactive and growth-oriented approach, ultimately improving collective effectiveness.<br>Believe in your ability to influence events, overcoming obstacles and uncertainties with determination, maintaining confidence, motivation, and resilience while continuously striving for excellence in patient care and teamwork.<br>Be aware of the impact of your behavior and actions on the well-being of the patient, the team, and the work environment, always promoting a mindset focused on continuous quality improvement in care.
6	Coping with Stressor	6.1 Coping with Stressors<br>6.2 Coping Strategies<br>6.3 Personal well-being	Endure long shifts while managing physical and mental stress, remaining calm during critical moments and maintaining focus under pressure.<br>Adapt to stressful situations by managing stress effectively and reflecting on each action to evaluate potential consequences, preventing complications or errors.<br>Utilize relaxation techniques to handle unforeseen problems and make swift decisions, ensuring clarity and control in challenging situations.<br>Identify creative and alternative solutions in advance, preparing for unexpected events and addressing them calmly to minimize anxiety for oneself and the team.<br>Take strategic breaks during lengthy interventions to manage stress and ensure sustained focus and performance.

			Be willing to ask for help when necessary, showing an understanding of when support is needed in stressful situations.<br>Manage personal stress by adopting techniques that foster emotional resilience and mental well-being.<br>Adopt coping strategies for stress management, eliminating stressors from your environment and employing relaxation practices during particularly demanding moments.<br>Employ relaxation techniques to prevent burnout, engaging in physical, mental, and emotional self-care regularly to uphold overall well-being.<br>Promote personal health by managing stress with proactive strategies that support both mental and physical wellness.<br>Engage in activities that provide pleasure, ensuring balance and well-being both inside and outside of work.
7	Resilience and Fatigue Management	7.1 Resilience<br>7.2 Reliability and Perseverance<br>7.3Fatigue Management<br>7.4 Workload Management	Manage critical situations without losing control, demonstrating determination and the ability to cope with rapid and unforeseen changes calmly and effectively.<br>Adapt to difficulties arising from changes with flexibility, constancy, and a positive attitude, ensuring that challenges do not derail progress.<br>Demonstrate resilience by keeping commitments and maintaining focus on goals, even when faced with adversity or difficult circumstances.<br>Maintain steadfast motivation and perseverance, pursuing your objectives with firmness and dedication, regardless of obstacles or setbacks.<br>Stick to your own commitments and remain determined, even when pressure, adversity, or failure threaten to derail your own progress.<br>Demonstrate resilience by reviewing your plans and adjusting them as necessary to stay on track toward achieving of own goals.<br>Cope effectively with inconveniences, obstacles, and difficulties arising from changes, while maintaining motivation and focus on long-term success.<br>Recognize the causes and effects of fatigue, and understand how it impacts both own well-being and the functioning of the team in high-risk environments.

			Protect both your own and others' well-being by actively managing fatigue and preventing its negative impact on performance in critical settings.<br>Endure long shifts and manage physical and mental stress effectively, recovering quickly after complex interventions and maintaining performance under pressure.<br>Manage workload by recognizing the signs of fatigue and taking necessary steps to recover quickly, ensuring that you remain productive and focused throughout the workday.
8	Leadership	8.1 Leadership<br>8.2 Ability to delegate<br>8.3 Be exemplary<br>8.4 Taking Responsibility	Exercise leadership by mobilizing authority without authoritarianism, avoiding lax behavior, and using authority only when necessary.<br>Maintain focus on achieving results, ensuring work proceeds effectively and resources are managed safely and consistently.<br>Be open to change, ready to review decisions when necessary, and adaptable to different situations and group needs.<br>Interpret situations clearly, anticipate future developments, and plan intervention strategies with the team, considering the opinions of all members.<br>Consult with more experienced colleagues when necessary and pass on knowledge to less experienced colleagues without humbling them.<br>Provide support and motivation to the team, recognize individuality, capitalize on the strengths of each member, and offer growth opportunities.<br>Use fair conflict resolution methods, facilitate communication and discussion among team members, and foster a positive and collaborative work environment.<br>Assume the formal leadership role of the operating room nursing team, actively participate in organizational decisions, and promote interprofessional collaboration.<br>Coordinate the team’s work, distribute tasks fairly based on available resources, and closely monitor task execution, ensuring appropriate control according to each member's professional level.<br>Delegate tasks and responsibilities effectively, assigning appropriate activities to the team and also, to the support staff, while assisting team members who may be struggling.<br>Lead by example, demonstrating how to manage critical situations, adhering to shared principles, and taking responsibility for both personal and team actions.

9	Coping with Emotions	9.1 Coping with emotions<br>9.2 Emotional management<br>9.3 Empathy<br>9.4 Emotional contagion	Understand and manage your own emotions, avoiding being overwhelmed by unpleasant feelings and maintaining control in difficult situations, ensuring focus on the safety of the person assisted.<br>Avoid reacting impulsively and manage anxiety or frustration that may arise in critical moments, promoting a serene environment and preventing burnout.<br>Manage your emotional responses in high-stress situations, maintaining attention and mental clarity, and recognizing when emotional intensity is high without being overwhelmed.<br>Promote emotional literacy within the team, encouraging shared understanding of emotions and developing emotional skills to improve communication with all healthcare providers.<br>Be self-motivated to understand your own emotions and foster mutual respect, employing strategies to motivate both yourself and others to manage emotions effectively.<br>Make rational decisions, rationalizing your responses before reacting and modulating emotional reactions to maintain balance in unforeseen contexts.<br>Foster a serene organizational climate and encourage the use of emotional intelligence among nurses, facilitating a collaborative and cohesive work environment.<br>Understand and recognize the emotions of the team, cultivating empathy by acknowledging both your own emotions and those of others to promote a supportive environment.<br>Engage in building emotionally authentic relationships and promote an emotionally safe workplace, ensuring that team members feel supported and valued.
10	Task and Time Management	10.1 Task Management<br>10.2 Time Management<br>10.3 Organization Anticipatory thought	Accurately plan surgery and anesthesiological activities, considering possible complications and organizing the materials and space required for each procedure.<br>Manage tasks according to priorities and available resources, ensuring the most efficient use of time, materials, and equipment in the surgical and anesthesiological workflow.<br>Plan activities effectively, setting clear priorities and making use of available resources to optimize both space and time, while adhering to established standards and guidelines.<br>Participate in evidence-based activity planning, organizing resources and materials in a functional way to ensure smooth operations and efficient work processes.<br>Anticipate potential problems before they arise, always preparing alternative plans in case of complications and reducing the margin of error through proactive thinking.

			Demonstrate initiative by preparing for possible complications and planning solutions to common problems, taking preventive action to reduce risks during surgery and anesthesia.<br>Organize the steps of the surgical and anesthesiological procedures efficiently, ensuring that activities flow smoothly without unnecessary delays or loss of time.<br>Prioritize tasks to optimize the pace of work and maintain a balance between speed and quality, ensuring that the most important activities are completed first, especially during urgent situations.<br>Maintain a focus on time productivity, ensuring that the necessary time is devoted to critical phases of care, while managing workflow efficiently to avoid slowing down activities.
11	Ethical and sustainable thinking	11.1 Sustainability<br>11.2 Ensuring environmental health<br>11.3 Ethical awareness<br>11.4 Advocacy<br>11.5 Legal, ethical and deontological accountability	Manage waste such as gases, drugs, sharps, and infectious materials in an environmentally friendly manner, actively participating in environmental sustainability efforts and assessing the environmental impact of new technologies, equipment, and resources introduced in the operating room.<br>Reduce regulated medical waste, recycle materials, diversify solid waste, and reuse or reprocess operating room materials whenever possible, including replacing disposable items with reusable alternatives to minimize environmental impact.<br>Promote awareness of the environmental impact of surgical and anesthesiological practices, adopting strategies to reduce waste, such as removing rarely used instruments from surgical sets and replacing disposable instruments with more sustainable options.<br>Ensure that all materials used in the operating room are selected with sustainability in mind, and help implement environmental sustainability programs by preventing anesthetic gas and drug pollution, working to reduce the environmental footprint of the entire surgical process.<br>Apply ethical principles to clinical-care practice, respecting the personal and moral values of the person being assisted, their family, caregivers, and all team members involved in care. Formulate expected outcomes in synergy with the patient, taking ethical principles into consideration.<br>Contribute to the creation and maintenance of an ethical environment within the operating room, ensuring safe, high-quality care is provided in line with ethical standards and reflecting on the consequences of decisions and actions.<br>Recognize that personal moral values can influence professional decisions and interventions, demonstrating a thorough understanding of ethical principles and applying them when formulating expected outcomes or making decisions about care.

			Intervene in the face of illegal, incompetent, or compromised practices to protect the person assisted, safeguarding their privacy and dignity throughout the perioperative and perianesthetic process.<br>Uphold patient confidentiality and ensure their well-being is always prioritized.<br>Support the person in making informed decisions about their care, ensuring their privacy, dignity, and rights are respected. Advocate for fairness and equity in the treatment of all individuals, promoting behaviors that demonstrate integrity, honesty, accountability, and commitment.<br>Adhere to the code of ethics, respect the dignity and privacy of the person, and approach the person being assisted with authenticity and accessible language, ensuring clear communication and professional conduct at all times.<br>Take responsibility for contributing to the dignity and integrity of the nursing profession, intervening against unethical behaviors, responding appropriately to concerns about unsafe practices, and adhering to safety, security, and data processing protocols.<br>Ensure consistent and safe management of resources, promoting a culture of ethical behavior and integrity, and intervening when necessary to ensure the protection of patients and the ethical standards of the healthcare environment.
12	Adaptation to the context	12.1 Cultural adaptability<br>12.2 Adaptation to the context<br>12.3 Adaptability Cultural Respect	Collaborate effectively with different teams, adjusting to their dynamics and working harmoniously with diverse individuals.<br>Interact with patients in a sensitive and respectful way, always considering their unique experiences, expectations, and individual preferences.<br>Adapt communication styles, behavior, and approach to meet the diverse cultural needs of patients, considering cultural differences in the healthcare setting.<br>Respect and accommodate cultural differences, including language barriers, by ensuring all team members can work cohesively, despite cultural or linguistic diversity.<br>Recognize and respect the values, beliefs, and traditions of patients and colleagues, promoting inclusivity and understanding within the healthcare environment.<br>Ensure fair, culturally sensitive care for all patients by acknowledging how cultural background influences their perception of illness, treatment, suffering, and healthcare practices in general.<br>Respond appropriately to patients' specific cultural needs, such as religious beliefs, preferences regarding gender, or physical contact during surgery or anesthesia.

			Demonstrate flexibility and adaptability by using different tools or technologies and adjusting to new roles, responsibilities, or changing situations within the operating room or healthcare setting.<br>Understand and respect the cultural differences between people of various backgrounds, traditions, and beliefs, and adjust care practices accordingly to create a respectful and inclusive environment for both patients and colleagues.
13	Critical Thinking	13.1 Critical Thinking<br>13.2 Open and critical mindset	Gather information objectively and distinguish between opinions and actual facts, ensuring that decisions are based on evidence rather than assumptions.<br>Reflect on every action and assess the possible consequences to ensure that decisions align with desired outcomes and quality standards.<br>Observe situations around and analyze them quickly to make informed decisions in real-time, ensuring an effective response to dynamic situations.<br>Formulate personal judgments based on solid evidence and ask relevant questions to address any problems or doubts that may arise.<br>Evaluate your own performance based on available evidence, reflecting on effectiveness and identifying areas for quality improvement.<br>Apply logic to draw conclusions, ensuring that judgments are well-supported and reasoned, and act based on concrete, evidence-based information.<br>Recognize your own limitations and biases, being aware that these can influence your judgment and impact decision-making processes.<br>Encourage critical discussion within the team to stimulate analytical thinking and challenge assumptions, leading to more robust decision-making.<br>Demonstrate intellectual curiosity by asking thoughtful questions and reflecting on your actions to identify areas for improvement in both individual and team practice.<br>Be willing to reconsider your opinions in light of new evidence, fostering an open and critical mindset that adapts to new information and experiences.
14	Learning through experience	14.1 Reflect<br>14.2 Learning to learn	Regularly reflect on both your successes and failures to identify key lessons that can enhance your future performance and create value for yourself and your team.<br>Continuously assess whether you have achieved the objectives you set, analyze the reasons for success or failure, and use this insight to improve your future goal-setting and execution.

		14.3 Learning from experiences	Provide honest and constructive feedback on both your own and others' successes and failures, and actively learn from the feedback you receive to drive personal and team growth.<br>Seek opportunities to enhance your own strengths and reduce areas for improvement, actively working on both personal and team development.<br>Keep your knowledge up to date by staying informed about advancements in clinical practice, ensuring you are always prepared to provide the best care.<br>Foster a work environment that embraces a culture of learning, encouraging both yourself and others to continuously improve and grow professionally.<br>Exhibit intellectual curiosity by asking insightful questions, challenging assumptions, and seeking new learning opportunities to deepen your understanding of your field.<br>Commit to lifelong learning through continuing education, professional development, and personal growth, ensuring you remain adaptable and capable throughout your career.
15	Data, Information and Digital Content Management	15.1 Research, Evaluate and Manage Digital Content<br>15.2 Manage data, information, and digital content	Engage in the digital literacy process by developing skills to navigate, assess, and evaluate digital environments for relevant data and content.<br>Perform routine and well-defined searches to locate data, information, and digital content, ensuring that your search process is structured and efficient.<br>Organize and plan digital searches in a way that helps you retrieve the most relevant and accurate data, information, and content.<br>Take a proactive and systematic approach when using digital technologies, applying them effectively for research and content management.<br>Navigate, search, and filter through digital data, information, and content to find the most relevant resources in digital environments.<br>Analyze, compare, interpret, and evaluate the quality of digital data, information, and content to ensure its reliability and usefulness.<br>Adapt to digital advances in perioperative and perianesthesiological clinical care, incorporating new technologies into your daily practice.<br>Collaborate and share information effectively with team members through digital technologies, ensuring smooth communication and coordination.
